# Concomitant Clonal *CBFB*::*MYH11* and *PDGFRB* Fusions in a Case of *De Novo* Acute Myeloid Leukemia

**DOI:** 10.3390/hematolrep18020024

**Published:** 2026-03-23

**Authors:** Qiliang Ding, Natasha E. Lewis, Cody J. Artymiuk, Renee M. Olson, Rong He, Rhett P. Ketterling, David S. Viswanatha, Patricia T. Greipp, Cinthya J. Zepeda Mendoza

**Affiliations:** 1 Division of Laboratory Genetics and Genomics, Department of Laboratory Medicine and Pathology, Mayo Clinic, Rochester, MN 55905, USA; 2 Division of Hematopathology, Department of Laboratory Medicine and Pathology, Mayo Clinic, Phoenix, AZ 85054, USA; 3 Division of Hematopathology, Department of Laboratory Medicine and Pathology, Mayo Clinic, Rochester, MN 55905, USA

**Keywords:** acute myeloid leukemia, inv(16), CBFB::MYH11, MLN-TK, PDGFRB, GOLGA4

## Abstract

**Background:** Acute myeloid leukemia (AML) with *CBFB*::*MYH11* fusion and myeloid/lymphoid neoplasms with eosinophilia and tyrosine kinase gene fusions (MLN-TK) are genetically defined and typically mutually exclusive entities. **Case Presentation:** We report a unique case of *de novo* AML harboring two clonal, transcriptionally active class-defining fusions: *CBFB*::*MYH11* and *GOLGA4*::*PDGFRB*. A 61-year-old woman presented with leukocytosis with neutrophilia, eosinophilia, and monocytosis; circulating blasts; and a markedly hypercellular marrow. Cytogenetic analysis revealed inv(16)(p13.1q22) and t(3;5)(p21;q32) in all 20 metaphases, and RNA sequencing confirmed expression of both *CBFB*::*MYH11* and *GOLGA4*::*PDGFRB* fusions. In addition, an oncogenic *WT1* frameshift variant was identified. Hematopathologic findings were largely consistent with AML with *CBFB*::*MYH11* fusion but exhibited features reminiscent of *PDGFRB*-rearranged MLN-TK. The patient achieved complete remission following the standard 7 + 3 induction chemotherapy regimen for AML with gemtuzumab ozogamicin. **Conclusions:** This case illustrates the diagnostic challenges posed by concomitant class-defining alterations in hematologic neoplasms and underscores the importance of integrated genomic assessment.

## 1. Introduction

Acute myeloid leukemia (AML) is an aggressive hematologic neoplasm caused by the clonal expansion of immature myeloid precursors. Genetic alterations are central to AML pathogenesis and underpin both disease classification and prognostication [1,2,3]. Core-binding factor AML (CBF-AML) represents a favorable-risk group driven by either *RUNX1*::*RUNX1T1* or *CBFB*::*MYH11* fusions. Disruption of the CBF transcription factor complex impairs hematopoietic maturation and promotes leukemogenesis [4]. In cases harboring *CBFB*::*MYH11*, the fusion protein exerts a dominant-negative effect on wild-type CBFB, thereby dysregulating the transcription of RUNX1 target genes [5].

In parallel, current classification schemes recognize myeloid/lymphoid neoplasm with eosinophilia and tyrosine kinase gene fusions (MLN-TK) as a distinct entity. These neoplasms are driven by constitutive activation of tyrosine kinases, including *PDGFRA*, *PDGFRB*, *FGFR1*, and *JAK2*, among others [2,3], resulting in dysregulated signaling and proliferation. Clinically, they typically present as chronic myeloid neoplasms with eosinophilia but may manifest directly in blast phase with ≥20% blasts. The introduction of tyrosine kinase inhibitors (TKIs) has markedly improved outcomes in these patients [6].

The coexistence of two class-defining oncogenic drivers, particularly gene fusions, within the same hematologic neoplasm is exceedingly rare [7]. To our knowledge, there have been no prior reports of concomitant CBF-AML and MLN-TK-defining fusions in hematologic neoplasms. Herein, we describe a case of *de novo* AML harboring concomitant *CBFB*::*MYH11* and *GOLGA4*::*PDGFRB* fusions within a single cytogenetic clone. The patient presented with features consistent with AML harboring a *CBFB*::*MYH11* fusion yet demonstrated additional atypical hematopathologic findings reminiscent of MLN-TK. Cytogenetic and RNA sequencing analyses confirmed that both fusions were transcriptionally active and present in the same clone.

## 2. Materials and Methods

Genomic testing was performed in a CLIA-certified, CAP-accredited clinical laboratory (Department of Laboratory Medicine and Pathology, Mayo Clinic, Rochester, MN, USA). Cytogenetic studies included chromosome analysis (karyotyping), interphase FISH, and sequential metaphase FISH. For chromosome analysis, the bone marrow specimen was placed in unstimulated suspension culture (Chang Medium BMC; Fujifilm, Santa Ana, CA, USA) for 24 and 48 h at 37 °C and 5% CO_2_. Cultured cells were harvested using automated or manual methods [8]. Slides were prepared using a CDS-5 Cytogenetic Drying Chamber (Thermotron, Holland, MI, USA), dried at 65 °C overnight, stained by the GTL method, and scanned on the Metafer AutoCapt slide-scanning system (version 4.4.130; MetaSystems, Altlussheim, Germany) using an Axio Imager 2 microscope (ZEISS, Oberkochen, Germany). Subsequently, metaphases were analyzed using the MetaSystems Ikaros software (version 6.3.15.2).

For interphase FISH, slides were prepared from a direct harvest of the bone marrow specimen without culture. Clinically validated probes targeting common genetic alterations in AML (dual-fusion probes for *RUNX1/RUNX1T1*, *PML/RARA*, *CBFB/MYH11* and a break-apart probe for *KMT2A*) and MLN-TK (a tricolor-fusion probe for *FIP1L1/CHIC2/PDGFRA* and break-apart probes for *PDGFRB*, *FGFR1*, *JAK2*, *ABL1*, *FLT3*) were applied. The probes were developed in-house or obtained from Abbott (Abbott Park, IL, USA) or Agilent (Santa Clara, CA, USA). Denaturation was performed using a ThermoBrite system (Abbott, Abbott Park, IL, USA) at 75 °C for 5 min. Slides were then incubated in a humidified chamber at 37 °C for approximately 4–8 h to allow hybridization. Post-hybridization washes were performed manually or using the VIP2000 system (Abbott, Abbott Park, IL, USA) to remove excess probe. Slides were counterstained with 10 µL of 10% DAPI and coverslipped. Automated image acquisition was performed using the MetaSystems Metafer MetaCyte software (version 4.4.130) and a ZEISS Axio Imager 2 fluorescence microscope, followed by manual scoring using the MetaCyte software. Alternatively, slides were reviewed and scored manually using a DM6 B fluorescence microscope (Leica, Wetzlar, Germany). FISH scores and images were stored using the MetaSystems Neon software (version 1.4.146).

For sequential metaphase FISH, a metaphase previously analyzed by karyotyping was selected, and the corresponding GTL-stained slide was destained using NP-40, ethanol, and formaldehyde. The slide then underwent denaturation, hybridization, and DAPI counterstaining as described above. The targeted metaphase was then manually analyzed using a Leica DM6 B fluorescence microscope.

A 50-gene targeted RNA sequencing panel was also performed [9]. RNA was extracted from the bone marrow specimen using the Maxwell^®^ RSC simplyRNA Blood kit (Promega, Madison, WI, USA), converted to double-stranded cDNA, and purified. Sequencing library was prepared using the KAPA HyperPrep kit (Roche, Basel, Switzerland). Target enrichment was performed with xGen capture probes (Integrated DNA Technologies, Coralville, IA, USA) targeting the coding exons of the 50-gene panel. Paired-end sequencing was carried out on a NovaSeq 6000 instrument (Illumina, San Diego, CA, USA) with the goal of 20 million reads per sample. Fusion detection used a consensus-based approach (MetaFusion) [10] that integrated results from three fusion callers: Arriba [11], STAR-Fusion [12], and MAP-RSeq [13].

## 3. Case Presentation

**Figure 1:**
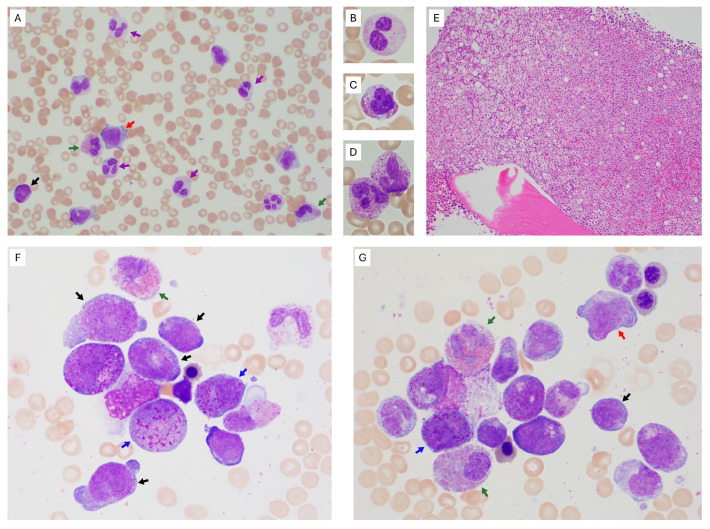
Peripheral blood smears (**A**–**D**), bone marrow core biopsy (**E**), and bone marrow aspirate smears (**F**,**G**) from the patient. (**A**) Peripheral blood smear at 500× magnification, showing circulating blast (black arrow) and monoblast/promonocyte (red arrow), monocytosis, morphologically atypical eosinophils (hypo or uneven cytoplasmic granulation, nuclear hyperlobulation; green arrows), and neutrophils with dysgranulopoiesis (cytoplasmic hypogranulation, nuclear hypolobation; purple arrows). (**B**–**D**) Peripheral blood smears at 1000× magnification, showing a hypogranular and hypolobated neutrophil (panel **B**), a morphologically atypical eosinophil with uneven cytoplasmic granulation, cytoplasmic vacuoles, and nuclear hyperlobulation (panel **C**), and an atypical mature myeloid cell with somewhat scant cytoplasmic eosinophilic and basophilic granules of unclear lineage, likely an abnormal eosinophil (panel **D**). (**E**) Bone marrow core biopsy at 100× magnification, showing markedly hypercellular marrow with increased myelomonocytic and eosinophilic elements with left-shifted maturation. (**F**,**G**) Bone marrow aspirate smears at 1000× magnification, showing blasts (black arrows) and a promonocyte (red arrow in panel **G**), increased maturing monocytes (panel **G**), mature eosinophils with atypical morphology (cellular enlargement, hyper- and hypo-lobated nuclei, uneven cytoplasmic granulation; green arrows), and abnormal eosinophil precursors with large purple cytoplasmic granules (blue arrows).

A 61-year-old woman presented to the emergency department with a three-week history of coughing, nausea, vomiting, weakness, and fatigue. Complete blood count (CBC) revealed leukocytosis (32.3 × 10^9^/L, reference: 3.4–9.6 × 10^9^/L), normocytic anemia (hemoglobin: 8.4 g/dL, reference: 11.6–15.0 g/dL; mean corpuscular volume: 97.3 fL, reference: 78.2–97.9 fL), and thrombocytopenia (77 × 10^9^/L, reference: 157–371 × 10^9^/L). Review of the peripheral blood smear (Figure 1A–D) further showed neutrophilia, eosinophilia, monocytosis, and circulating blasts and promonocytes (blast equivalents, 8% in total; Figure 1A). Subsets of neutrophils (Figure 1B), eosinophils (Figure 1C,D), and monocytes were observed with atypical morphology. Bone marrow core biopsy (Figure 1E) and aspirate smears (Figure 1F,G) demonstrated a markedly hypercellular marrow (>95%) with a marked increase in neutrophilic, eosinophilic, and monocytic elements. Myeloid maturation was left-shifted with increased blasts and promonocytes (31% in total; Figure 1E). Erythroid precursors were significantly reduced and showed occasional dyspoiesis. Megakaryocytes were reduced and showed no significant atypia. Detailed CBC data and bone marrow findings at AML diagnosis are provided in the Supplementary Materials.

**Figure 2:**
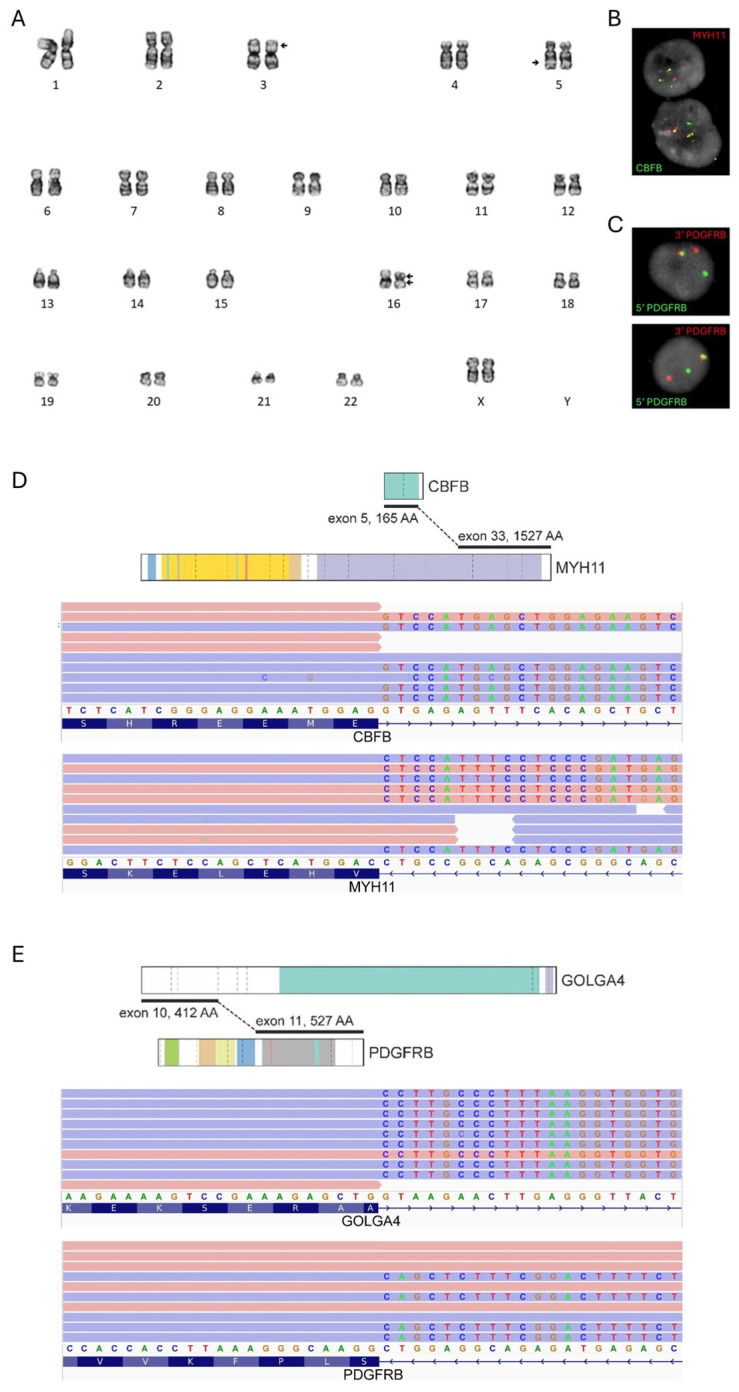
Diagnostic genetic studies. (**A**) Unstimulated bone marrow chromosome analysis demonstrated a 46,XX,t(3;5)(p21;q32),inv(16)(p13.1q22) karyotype in all 20 metaphases. Arrows: rearrangements. (**B**) Dual-fusion FISH confirmed *CBFB*::*MYH11* fusion (signal pattern: 1 red, 1 green, 2 fusion) in 71% of nuclei. The two fusion signals correspond to *CBFB*::*MYH11* and the reciprocal *MYH11*::*CBFB* fusion; the single red and green signals denote intact *MYH11* and *CBFB,* respectively. (**C**) Break-apart FISH revealed 5′ and 3′-*PDGFRB* separation (signal pattern: 1 red, 1 green, 1 fusion) in 84% of nuclei. The fusion signal corresponds to the intact *PDGFRB*; the single red and green signals represent separated 3′-end and 5′-end of *PDGFRB*, respectively. (**D**,**E**) RNA sequencing confirmed that both *CBFB*::*MYH11* (panel **D**) and *GOLGA4*::*PDGFRB* (panel **E**) were transcriptionally active. Fusion schematics were generated using ProteinPaint [14].

The patient had no known history of myelodysplastic syndrome (MDS) or myeloproliferative neoplasm (MPN). In addition, CBC results from approximately 1 year and 3.5 years prior to AML presentation were unremarkable, altogether supporting a diagnosis most consistent with *de novo* AML. Diagnostic bone marrow chromosome analysis revealed inv(16)(p13.1q22) in all 20 metaphases (Figure 2A), and *CBFB*::*MYH11* fusion was confirmed by a dual-fusion FISH probeset (71% of 200 nuclei, normal threshold: <4%; Figure 2B). Intriguingly, all 20 metaphases from the chromosome study also harbored a concomitant t(3;5)(p21;q32) (Figure 2A). Break-apart FISH testing of *PDGFRB* (at 5q32) was abnormal, showing 5′ and 3′ signal separation in 84% of 100 nuclei (normal threshold: <15%; Figure 2C). Sequential metaphase FISH analysis localized the 5′ *PDGFRB* locus to 3p21, consistent with the identified 3;5 translocation and suggestive of a functional *PDGFRB* rearrangement. Targeted RNA sequencing detected *CBFB*::*MYH11* and additionally identified *GOLGA4*::*PDGFRB*, confirming the presence of two transcriptionally active oncogenic fusions (Figure 2D,E). A myeloid next-generation sequencing panel additionally detected an oncogenic *WT1* frameshift variant, NM_024426.2:c.934_938dup, p.Ala314Glyfs*69, at 43% variant allele frequency. No additional oncogenic alterations were identified.

The patient underwent induction chemotherapy with seven days of cytarabine and three days of idarubicin (“7 + 3”), along with a single dose of gemtuzumab ozogamicin. She also received one dose of intrathecal cytarabine for central nervous system prophylaxis. Twenty-three days following the conclusion of induction, she achieved complete remission with negative minimal residual disease (MRD) by flow cytometry. Concurrent real-time quantitative reverse transcription PCR (qRT-PCR) demonstrated near- deep molecular remission, with *CBFB*::*MYH11* transcript detected at 3 per 10,000 *ABL1* copies (0.03%).

## 4. Discussion

Despite harboring a *PDGFRB* fusion, our patient’s presentation was most consistent with *de novo* AML with *CBFB*::*MYH11*. Key observations included increased blasts with monocytic differentiation, increased eosinophils and eosinophil precursors in the bone marrow, as well as eosinophil precursors with abnormally large dark purple cytoplasmic granules. There was no prior history of MDS, MPN, eosinophilia, or monocytosis. In contrast, in patients with *PDGFRB*-rearranged MLN-TK, eosinophilia and monocytosis are frequent (50–80% and 30–90%, respectively), with only about 15% present initially in blast phase [6]. Nonetheless, this case showed several features unusual for AML with *CBFB*::*MYH11* but reminiscent of *PDGFRB*-rearranged MLN-TK. These included peripheral eosinophilia at presentation, eosinophils with atypical morphology, neutrophils with dysgranulopoiesis, and mature myeloid cells of unclear lineage with scant cytoplasmic eosinophilic and basophilic granules.

*GOLGA4* (also known as golgin-245 and p230) encodes golgin subfamily A member 4, a coiled-coil protein that associates with the trans-Golgi network and supports retrograde membrane traffic to the Golgi. It also facilitates centralized Golgi positioning; when golgin-245 is depleted, Golgi elements disperse into “mini stacks” at the cell periphery [15]. Moreover, golgin-245 contributes to stress-induced autophagosome biogenesis [16]. Mouse and human data indicate that *GOLGA4* is highly expressed in hematopoietic cells [17], potentially enabling robust expression of the *GOLGA4*::*PDGFRB* fusion. In our patient, the fusion protein removed the extracellular ligand-binding domain of *PDGFRB* while preserving the tyrosine kinase domain (Figure 2E), consistent with the typical structure of oncogenic fusions involving *PDGFRB* [18]. The *GOLGA4*::*PDGFRB* fusion has previously been documented in two patients, both with MPN with eosinophilia, a phenotype that would likely be classified as MLN-TK under current frameworks. Both patients were responsive to TKI (imatinib) therapy [19].

It is exceptionally rare for two class-defining fusions to co-occur in hematological neoplasms [7], and this *CBFB*::*MYH11* and *PDGFRB* fusion combination has not been described in acute leukemia. In previously reported cases with dual class-defining oncogenic drivers, one alteration is often subclonal, reflecting the emergence of secondary events during disease evolution; an example of this is a recently described subclonal *STRN3*::*PDGFRB* fusion in an *NPM1*-mutated AML [20]. Among fusions reported to co-occur with *CBFB*::*MYH11*, *BCR*::*ABL1* is the most frequently documented, although only a few cases exist [5,7,21,22,23,24]. Most of these represent chronic myeloid leukemia (CML) progressing to blast phase, with *BCR*::*ABL1* preceding *CBFB*::*MYH11*. Rare cases of *de novo* AML in which *CBFB*::*MYH11* precedes *BCR*::*ABL1* or both occur simultaneously have also been described, and these cases are generally associated with an aggressive clinical course [5,21]. Our analyses showed no clear evidence of subclonal *CBFB*::*MYH11* or *GOLGA4*::*PDGFRB* fusion populations. Analysis of 96 available metaphases revealed both abnormalities co-occurred in 87 metaphases, while the remaining nine metaphases were apparently normal. To directly demonstrate that both fusions were present in the same cell population, we performed sequential FISH on a metaphase karyotyped as harboring both inv(16)(p13.1q22) and t(3;5)(p21q32). Using a *PDGFRB* break-apart probeset, we observed 5′- and 3′-*PDGFRB* signal separation, thereby confirming that the *CBFB*::*MYH11* fusion [from inv(16)] and the *PDGFRB* rearrangement [from t(3;5)] occurred within the same cytogenetic clone. While we cannot rule out one abnormality arising before the other, the combined clinical and genetic picture suggests that this neoplasm is most consistent with AML with *CBFB*::*MYH11*, albeit with morphologic features likely influenced by the concomitant *GOLGA4*::*PDGFRB* fusion.

In a study of 8226 patients with myeloid or lymphoid leukemia, only 25 (0.3%) harbored dual gene fusions [7]. When dual fusions are identified, they often follow a cooperative model of leukemogenesis: a Class I alteration producing constitutive kinase activation (e.g., *BCR*::*ABL1*) paired with a Class II alteration that disrupts hematopoietic differentiation (e.g., *CBFB*::*MYH11* or *RUNX1*::*RUNX1T1*). This combination satisfies the two-hit hypothesis, combining proliferative signaling with a maturation block [25]. In addition to the dual fusions, our patient had an oncogenic *WT1* frameshift variant. The mechanism of *WT1* loss-of-function variants in oncogenesis remains incompletely understood but is thought to involve, at least in part, epigenetic dysregulation [26]. The *WT1* variant may contribute to unfavorable risk, given its association with poor prognosis in AML [27], although its impact in the context of concomitant class-defining fusions remains unclear.

Lastly, the *PDGFRB* fusion may suggest potential sensitivity to TKIs, e.g., imatinib, which are highly effective in MLN-TK. Because this patient’s presentation was most consistent with AML with *CBFB*::*MYH11*, initial treatment followed established AML induction protocols without TKI administration, and the patient had achieved complete remission and MRD negativity by flow cytometry following induction. While initial AML-directed induction therapy achieved complete remission, the coexisting *PDGFRB* fusion may inform consideration of future salvage or maintenance approaches (should they become necessary). At relapse, a genetic re-evaluation would be necessary to assess clonal evolution and identify actionable targets. If the *PDGFRB* fusion is present at relapse, TKI may be considered as part of the cytoreduction therapy prior to allogeneic hematopoietic stem cell transplant (as recommended by the European LeukemiaNet for relapsed AML patients [1]). Alternatively, if transplant is not feasible, TKI may be considered for disease control. Nonetheless, it is critically important to point out that the role of TKI in improving outcomes in dual-fusion hematologic neoplasms remains speculative and uncertain; the current evidence consists only of isolated case reports describing favorable responses when TKIs were added during induction (e.g., [21]), and caution should be exercised on the generalizability of these reports.

## 5. Conclusions

Taken together, this case illustrates the diagnostic complexity that arises when two typically mutually exclusive, class-defining oncogenic drivers coexist, seemingly within the same clone. It emphasizes the importance of integrating clinical, hematopathologic, and genetic information to fully characterize such cases, which may show overlapping phenotypes that can blur diagnostic boundaries.

## Data Availability

All data supporting the findings of this case report are included within the article.

## Supplementary Materials

The following supporting information can be downloaded at: https://www.mdpi.com/article/10.3390/hematolrep18020024/s1, Detailed CBC data and bone marrow findings of the patient at AML diagnosis.
