# Supplementary material for: Concomitant Clonal CBFB::MYH11 and PDGFRB Fusions in a Case of De Novo Acute Myeloid Leukemia
Source: Hematol Rep. 2026 Mar 23;18(2):24. doi: 10.3390/hematolrep18020024 (PMC13116167; doi:10.3390/hematolrep18020024)
Supplement: Supplementary file 1 [file hematolrep-18-00024-s001.zip › hematolrep-4138542-supplementary.pdf]

Supplementary Materials
Concomitant clonal *CBFB::MYH11* and *PDGFRB* fusions in a case of *de novo* acute myeloid leukemia

The complete blood count results of the patient at AML diagnosis are as follows:

**Component**	**Value**	**Flag**	**Reference Range and Units**
Hemoglobin	8.4	Low	11.6–15.0 g/dL
Hematocrit	25.0	Low	35.5–44.9%
Erythrocytes	2.57	Low	3.92–5.13×10^{12}/L
Mean Corpuscular Volume (MCV)	97.3		78.2–97.9 fL
RBC Distribution Width	18.6	High	12.2–16.1%
Platelet Count	77	Low	157–371×10^{9}/L
Leukocytes	32.3	High	3.4–9.6×10^{9}/L

The bone marrow findings at AML diagnosis are as follows:
<u>Bone marrow aspirate smear and biopsy touch imprint:</u>

- **Bone marrow differential (% of total cells):**

Blasts/blast equivalents	31%
Promyelocytes	3%
Eosinophils	24%
Basophils	1%
Monocytes	8%
Lymphocytes	4%
Plasma cells	0%
Erythroid Precursors	7%
Neutrophils and Precursors	22%
Total Cells	500

- **Blasts/blast equivalents**: Increased. Blasts are intermediate to large with rounded to irregular nuclei, dispersed chromatin, distinct nucleoli, and scant to moderate cytoplasm. Promonocytes (blast equivalents) are increased.
- **Erythroid precursors**:
  - Quantity: Markedly reduced
  - Morphology: Full maturation with occasional dyspoietic forms
- **Myeloid precursors**:
  - Quantity: Myelomonocytic and eosinophilic elements are markedly increased
  - Morphology: Left-shifted maturation; eosinophil precursors are increased and show atypical large, dark purple cytoplasmic granules; occasional neutrophils with hypogranulation or atypical nuclear segmentation noted
- **Megakaryocytes**:
  - Quantity: Present
  - Morphology: No overt atypia
- **Lymphocytes**:
  - Quantity: Not increased
  - Morphology: No morphologic abnormalities
- **Plasma cells**:
  - Quantity: Not increased
  - Morphology: Unremarkable

Bone marrow clot and biopsy:

- **Cellularity**: Markedly hypercellular (>95%)
- **Erythroid precursors**:
  - Quantity: Markedly reduced
  - Morphology: Show maturation
- **Myeloid precursors:**
  - Quantity: Myelomonocytic and eosinophilic elements are markedly increased
  - Morphology: Left-shifted maturation
- **Megakaryocytes:**
  - Quantity: Reduced
  - Morphology: No overt atypia
- **Lymphocytes:**
  - Quantity: Not increased
  - Morphology: Scattered and in few minute, loose interstitial aggregates (best seen on the clot)
- **Plasma cells:**
  - Quantity: Not increased
  - Morphology: Normal morphology and distribution
- **Bony trabeculae**: Unremarkable
